# Image Recognition and Extraction of Students' Human Motion Features Based on Graph Neural Network

**DOI:** 10.1155/2022/6755053

**Published:** 2022-03-24

**Authors:** Jianguo Liu, Kai Ji, Yan Jing

**Affiliations:** School of Physical Education, Wuhan Sports University, Wuhan, Hubei 430079, China

## Abstract

In order to improve students' overall subhealth behavior, teenagers' physical health problems have attracted more and more attention. The state clearly requires students to increase the number and frequency of exercise in school. In order to study the physical changes in the process of students' sports and the impact on their health caused by a sports injury, a student human motion feature image recognition based on a graph neural network is proposed in this paper. This paper combines image recognition technology with graphic neural network management and uses image recognition technology to detect and track targets. It also analyzes the health changes of students in sports and the influencing factors of physical subhealth in classroom learning. The results show that image recognition technology can accurately analyze the process of cervical spine injury and sports injury in students' classroom activities. It provides accurate experimental data for analyzing students' physical health and effective suggestions for promoting students' healthy development. Compared with the traditional image recognition and analysis results, the advantage of using a graph neural network to manage the detection and tracking results is that a graph neural network is used to manage the detection and tracking results, and the visual expression of students' physical health test data is completed.

## 1. Introduction

With the rising demand of people's life, the quality of life is also undergoing benign changes. More and more people have begun to pay attention to health problems [1]. Due to the focus on the cultivation of teenagers, most countries began to pay attention to students' physical health and physical changes [2]. We analyze the current situation of physical health of contemporary primary and secondary school students and find that most of the students' physique has decreased. The reason may be that schools and educational staff do not pay attention to students' physical development, and students' family environment and students' own lack of health concept [3]. According to the survey, the overall development of primary and middle school students' comprehensive quality is lower, and the physical health problems are mainly reflected in overweight, height is not up to the age standard, subhealth, cervical spine damage, physical injury, and so on. Due to the complexity of the school environment and the imbalance of students' healthy development, we should ensure the premise of students' normal development and improve their own physical quality [4, 5].

Factors affecting students' physical health due to the deep influence of exam-oriented education, the original concept can not be changed immediately [6]. Therefore, it virtually increases the overall burden of students, resulting in neglect of physical health [7]. Secondly, the lack of sports equipment is also the reason affecting physical health. In school physical education, cultivating students to cultivate physical exercise is an important link in the development of moral character and comprehensive quality. Finally, there are family factors. Students' physical health problems are inseparable from their family environment [8, 9]. Most parents only know that they pay attention to students' academic development and do not pay attention to students' physical quality. Based on the above situation, we need to further study and analyze students' physical health [10]. Image recognition technology has developed rapidly and has been applied in many fields. Image recognition specifically includes character recognition, image recognition processing, object recognition, human tracking recognition, etc. [11, 12]. We found that the application of image recognition technology in paying attention to the changes in students' physical health has achieved effective research results. Due to the interference of various background factors in the recognition process, image recognition technology can also solve the problems such as judgment error in the process of manual recognition [13]. Human identification of information elements is inefficient and can not process a large number of image data. Image recognition technology can obtain the most intuitive data information through the analysis and ranking of a large number of data and store the data information. When people need to identify and analyze an aspect, they can directly obtain the changes of reference data [14].

This paper discusses and analyzes the changing factors of students' physical health. The innovative contributions of this paper are as follows: (1) Compared with the traditional recognition technology, we use the red marker aggregation region for local capture. This method greatly improves the accuracy and efficiency of image recognition. (2) This paper focuses on students' cervical health problems in classroom activities and analyzes the change trend of cervical spine through students' learning time and bow image acquisition. (3) The graph neural network algorithm is used to manage and distribute the information data after image recognition. This enables students' health data to be displayed intuitively and can be fed back to testers faster when storing data. The experimental results show that most students have physical and subhealth problems. Image recognition technology can quickly extract feature points in the classroom environment and other interference backgrounds and track and identify students' behavior targets.

This paper is mainly divided into three parts. The first part is to understand the advantages of image recognition technology and the development status of various countries and put forward the research content of this paper. The second part first studies the physical changes and injuries of students in the process of sports by using image recognition technology combined with graph neural network management data. Finally, it studies the subhealth problems and the changes of cervical and vertebral health produced by students in the process of learning in the classroom environment by using image recognition technology. The third part analyzes the results of the research on students' physical health problems after using image recognition technology and graph neural networks to manage data.

## 2. Related Work

Due to the call of the corresponding countries, most schools began to pay attention to the health problems of students. When analyzing and studying students' physical health, we can obtain students' health change data through character behavior tracking and image processing through image recognition technology [15]. Now the transmission speed of artificial image recognition technology is accelerating, which can meet the research needs of the medical field, education field, and military field. And gradually convert from the traditional two-dimensional image mode to multidimensional image mode [16, 17]. Finally, it is combined with virtual simulation technology to form an intelligent multidimensional image recognition technology. At present, image recognition technology has the advantages of large storage and rapid processing of graphic information to obtain feature points [18]. However, with the continuous in-depth exploration of human body detection, target tracking, motion tracking, and other functions, the traditional image recognition technology can not meet the processing and storage of a large amount of data [19]. Therefore, we need to add dynamic network management after graph neural network optimization to manage and store the information after identification and processing and finally realize the visualization of data results [20].

The United States is in a leading position in the research of image recognition technology. From the perspective of the traffic field, combined with vehicle recognition function, it improves the efficiency of traffic management [21]. In road environment detection, different types of roads and vehicle driving conditions are quickly analyzed through image recognition, then fed back the processing results to the traffic management system for road coordination and control.

The UK combines image recognition technology with cloud computing to realize image upload, processing, classification, and other functions [22, 23]. They will obtain the pixel values of the image for analysis and classification and conduct the image edge range test to determine the image size. Finally, the real contrast and similarity of images are calculated uniformly so as to judge the problems of duplicate images in cloud network platforms.

Germany mainly applies this technology to food safety. Due to the species of bacteria, the food image is small [24]. The background of the acquired image is most complex, so it can not accurately distinguish the key content and interference factors. This leads to low accuracy of image recognition. Therefore, fine-grained image recognition is proposed to analyze the strain style, which improves the accuracy of food safety detection.

China has applied image recognition technology in the field of industrial converter automation. It can realize the control and detection of tapping results [25]. The spectrum is analyzed by image recognition to improve the range and accuracy of the cutting area. Finally, realize automation and improve efficiency. They also applied this technology to analyze tunnel traffic on the expressway. It reduces the traffic accidents caused by terrain, light, traffic flow, and other factors in the tunnel scene. Based on the research status of the above countries, this paper proposes to study and analyze the students' physical health status and influencing factors by combining image recognition technology with graph neural network management technology.

## 3. Research on Students' Physical Health Problems in Classroom and Sports Based on Image Recognition Technology and Graph Neural Network Management

### 3.1. Research on the Changes of Students' Physical Health after Exercise Based on Image Recognition Technology and Graph Neural Network Management

**Figure 1:**
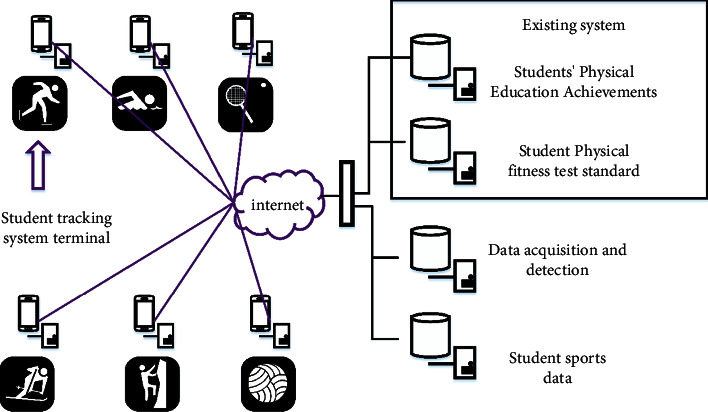
Structure diagram of student physical health system.

Subhealth means that the body has no obvious diseases, but the health state is not very good, and the physical condition is on the edge of health and disease. The main causes of adolescent students' subhealth are a heavy academic burden and great employment pressure. The social environment leads to the emergence of an inferiority complex, improves students' physical quality, pays more attention to physical health, and so on. The main causes of subhealth of young students and their sports countermeasures have a very important impact on the physical health of young students, which can effectively improve the enthusiasm of young people to exercise and improve their physical quality. Moreover, through the popularization of some subhealth knowledge, teenagers can pay more attention to subhealth and their own health. At present, many primary and middle school students generally have physical quality problems such as subhealth, and there are many influencing factors of physical health. With more and more students' myopia, heart injury, and sports injury, schools and researchers began to focus on the influencing factors and the healthy development of students. The traditional recognition technology has some problems, such as low success rate, long analysis time, and small storage environment. We propose an image recognition automatic tracking technology to analyze the movement changes and injuries of students in different intensity sports. After acquiring the image target of the student activity range, the target image of the student activity is extracted by segmenting the original image, and filtering and excluding the result to obtain the center distance position of the image. Finally, the pixel classification of motion action is carried out to establish the model. Before tracking and detecting the movement process, first deploy a student physical health system, as shown in Figure 1.

According to the function of the system in Figure 1, students' sports and other activities are detected and analyzed in real time. Wavelet transform is required to extract the target of student action image, which is expressed as follows:

(1) $$\begin{array}{c} A\left(i,j\right)\left(k,l\right)=\left\|B\left(i,j\right)\cdot C\left(k,l\right)\right\|, \end{array}$$

where *A*(*i*, *j*)(*k*, *l*) is the filtering result of the human body target image after being processed by the filtering network structure. *B*(*i*, *j*) is the moving image target and *C*(*k*, *l*) is the small filter combination. Next, you need to define the centralization of nonstationary moving images within the active range:

(2) $$\begin{array}{c} E_{pq}=\sum_{x}\sum_{y}{\left(x-\overset{-}{x}\right)}^{p}{\left(y-\overset{-}{y}\right)}^{q}D\left(x,y\right). \end{array}$$

In the formula, (*x*, *y*) is the central coordinate of the action image. In order to accurately analyze the image, four matrices need to be defined. They represent translation, rotation, scale invariance, etc. According to the matrix change results, calculate the normalized center distance of the human action diagram under different exercise intensity, and the formula is as follows:

(3) $$\begin{array}{c} \eta_{pq}=\frac{E_{pq}}{E_{00}^{\gamma }},\gamma =\frac{p+q}{2}+1,p+q=2,3,4... \end{array}$$

In this paper, the center distance of students' action images is analyzed by vector classification method. It is assumed that at any time, the pixel probability in the action image is expressed as follows:

(4) $$\begin{array}{c} p\left(G_{ij}^{t}\right)=\sum_{s=K,L}p_{s}\left(G_{ij}^{t}|\theta_{ij,s}^{t}\right). \end{array}$$

In the formula, *G*_*ij*_^*t*^ is the pixel in the target result graph at a certain time. *p*_*s*_(*G*_*ij*_^*t*^*|θ*_*ij*,*s*_^*t*^) represents the probability that image pixels exist before and after the environment. At any time, the pixel environment mixing model within the student's activity range can be expressed as follows:

(5) $$\begin{array}{c} p_{b}\left(G_{ij}^{t}\right)=\sum_{K}w_{ij,b,k}^{t}\times \eta \left(G_{ij}^{t},\mu_{ij,b,k}^{t},\sum_{ij,b,k}^{t}\right). \end{array}$$

In the formula, *K* is the number of pixel colors of the action image expressed by the hybrid model. *w*_*ij*,*b*,*k*_^*t*^ is the weight value in the action image background at any time. The weight value satisfies the following formula:

(6) $$\begin{array}{c} \sum_{k}w_{ij,b,k}^{t}=1. \end{array}$$

After getting the action background image, compare the difference between the number of action image frames of students under different intensity exercise and the background. The human motion image target is obtained, which is expressed as follows:

(7) $$\begin{array}{c} p_{u}\left(y\right)=I_{h}\sum_{i=1}^{n}k\left({\left\|\frac{y-x_{i}}{h}\right\|}^{2}\right)\delta \left[b\left(x_{i}\right)-u\right]. \end{array}$$

In the formula, *I*_*h*_ is the normalization factor, *u* is the target eigenvalue of the action image, and *y* is the central pixel of the image. According to the above formula, the student motion feature data are calculated and extracted, and finally, the student motion target model is constructed. Then, the physical data of athletes are analyzed, and the sports injury images of students are obtained. The motion injury image is converted to gray scale. Compared with traditional color images, byte representation can be performed according to pixels. The corresponding brightness is generated according to the bytes. When the three brightnesses are the same, it is a gray image. On the contrary, when the values are different, it is a color image. The gray value conversion formula is as follows:

(8) $$\begin{array}{c} \text{Gray}\left(i,j\right)=0.299\cdot R\left(i.j\right)+0.587\cdot G\left(i.j\right)+0.114\cdot B\left(i.j\right). \end{array}$$

**Figure 2:**
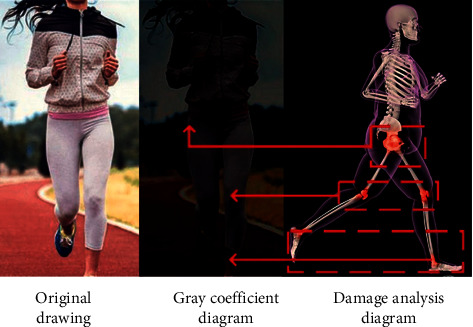
Comparison between the original image and gray image.

After conversion, the bitmap pixel representation of the image remains unchanged. Therefore, the above gray conversion is mainly to improve the damage identification effect. The comparison between the original image and gray image before and after conversion is shown in Figure 2.

It can be seen from Figure 2 that by comparing the original image with the gray coefficient image, it is concluded that the injury parts in sports are mainly reflected in the hip joint, knee joint, and ankle joint. Therefore, the analysis of students' physical health can clearly identify the location of sports injury and improve their health. In order to distinguish the damage of students' local activities and improve the accuracy, it is necessary to extract the features of local range structure. We need to carry out nonhealth positioning according to mathematical modeling and defining threshold. The expression formula is as follows:

(9) $$\begin{array}{c} E\left(C\right)=\left[aE_{in}\left(C\right)+\beta E_{es}\left(C\right)\right]\text{Gray}\left(i,j\right), \end{array}$$

where a and *β* represent weighted values, *E*_*in*_(*C*) and *E*_*es*_(*C*) represent external and internal values, respectively. After obtaining the damage range, the damage location is initialized and identified according to the transformation analysis method. After obtaining the contour range, a numerical matrix is established according to the number of pixels. To improve the accuracy of recognition position, the images need to be arranged as feature vectors. The formula is expressed as follows:

(10) $$\begin{array}{c} u=\frac{1}{m}\sum_{i=1}^{m}x_{i}\cdot E\left(C\right). \end{array}$$

In this paper, the eigenvectors are arranged in a decreasing way. After the arrangement, you need to select the nonzero vector, calculate the covariance matrix according to the following formula, and select 60% of the eigenvalue to save most of the damaged images.

(11) $$\begin{array}{c} u_{i}=A\frac{1}{\sqrt{\lambda_{i}}}x\cdot v_{i}\cdot \mu . \end{array}$$

Finally, all the moving image samples of students to be recognized are projected into the spatial model, and the projection coefficient is calculated according to the following formula:

(12) $$\begin{array}{c} y_{i}=U^{T}\cdot u_{i}. \end{array}$$

Then calculate the distance for all image samples to be recognized and training data according to the following formula. The selected minimum distance value sample is the preliminary identification result.

(13) $$\begin{array}{c} d\left(x,y\right)={\left[\sum_{i=1}^{n}{\left(x_{i}-y_{i}\right)}^{2}\right]}^{1/2}, \end{array}$$

where *n* represents the number of training samples and *d*(*x*, *y*) represents the European distance.

**Figure 3:**
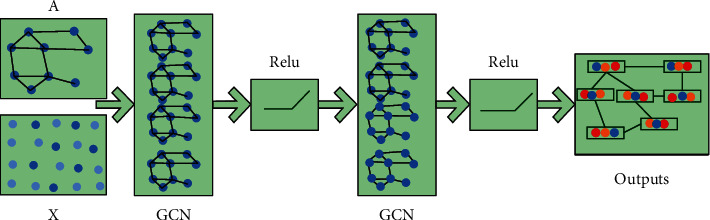
Figure structure diagram of neural network.

Through the above formula, the damage of students in the process of sports is calculated to detect physical health and other problems. In order to further analyze whether the students' overall physical health meets the standards, we input the training and identified data into the graph neural network management. Graph neural networks can extract features from the European spatial data calculated above so as to obtain the data we need. The structure of the neural network is shown in Figure 3.

As can be seen from Figure 3, the spectral domain graph convolution network and spatial domain are combined. Be able to recognize the signal of the diagram for processing. The graph convolution network is used in this paper. Compared with other graph neural network structures, it can process the updated feature points and has higher efficiency, more flexibility, and versatility. We define graph convolution as the product of signal and filter function, and the expression is as follows:

(14) $$\begin{array}{c} g_{\theta }^{*}x=Ug_{\theta }U_{x}^{T}. \end{array}$$

*g*_*θ*_ is the filter function and *x* is the signal of the image node. Finally, the eigenvalues of the function matrix are formed into a diagonal matrix for approximate processing, and the expression is as follows:

(15) $$\begin{array}{c} h^{\left(l\right)}=\sigma \left({\overset{-}{D}}^{1/2}\overset{-}{A}{\overset{-}{D}}^{1/2}h^{\left(l-1\right)}W^{\left(l-1\right)}\right. \end{array}$$

where *σ* is the nonlinear activation function and *W*^(*l* − 1)^ is the numerical matrix of graph convolution network. In this paper, the feature data after image recognition is managed by a graph neural network, which has faster operation efficiency and accuracy than the traditional recognition algorithm. It can accurately analyze the changes of students' physical health.

### 3.2. Research on Cervical Spine Health Problems of Students' Classroom Activities Based on Image Recognition Technology

**Figure 4:**
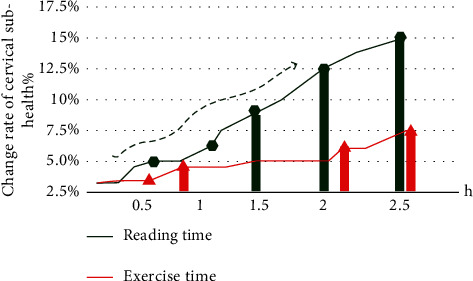
Comparison of the subhealth change rate of cervical spine between reading time and exercise time.

As an important way of processing image information in the computer field, image recognition technology plays different roles in people's life. Based on the image recognition technology, this paper analyzes the students' classroom activities and detects the students' overall physical health. This paper investigates the problems of students, such as cervical subhealth. The factors affecting the reading time and exercise time of the cervical spine were analyzed and discussed. The results showed that 60% of the students had mild cervical spondylosis and other complications caused by cervical vertebra.  Figure 4 shows the comparison of the change rate of cervical subhealth between students' reading time and exercise time.

**Figure 5:**
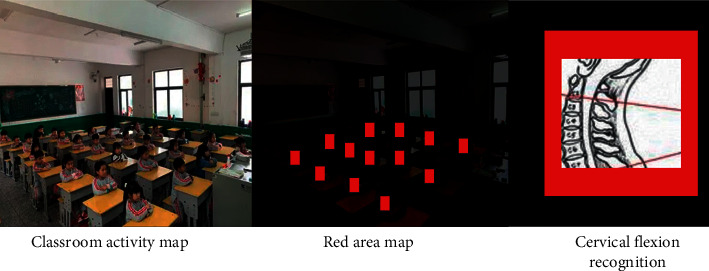
Experimental process and feedback results.

It can be seen from Figure 4 that most students spend two to three hours reading, and the impact on cervical subhealth is very significant. The increase of exercise time will reduce the change rate of cervical subhealth. The effect of the cervical subhealth change is the greatest between 1.5 hours and 2 hours. According to the above situation, we will detect the process of students in class and obtain the students' class state image at each time for recognition. The target color histogram model is used to convert the position of the feature box to locate the center position of the target in the current image. In this paper, the target tracking optimization algorithm camshift algorithm is used to identify the video area. Compared with the traditional tracking and recognition algorithm, it can improve the target tracking accuracy and effectively reduce the error. Facing the complex classroom environment, we can provide conditional feedback to the original algorithm according to the color feedback method. We extract the red pixels in the classroom area for color clustering to obtain the contour and center coordinates of the red target. The experimental process and feedback results are shown in Figure 5.

**Figure 6:**
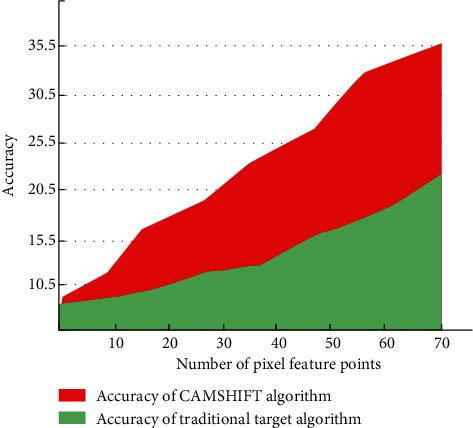
Comparison of accuracy between traditional target tracking algorithm and CAMSHIFT algorithm.

Students' goals can be tracked through local color feedback. Secondly, according to the needs of the experimental environment, students wear the same color uniforms for classroom activities. We mark the student's class clothes for easy detection. Mark the area between the head and the coat in the image, and use the above pixels to gather the target areas respectively. Eliminate the interference factors of black and environment so as to detect the changes of cervical spine bending during students' learning. Compared with traditional target tracking algorithms, the accuracy of camshift algorithm used in this paper is shown in Figure 6.

It can be seen from Figure 6 that the traditional target tracking and recognition algorithm can not improve the accuracy with the increase of image feature points. The camshift algorithm used in this paper can improve the detection accuracy in the case of a large number of pixel features. When we start the classroom monitoring mode to obtain students' local images, we analyze the local changes of students' cervical spine according to the image recognition technology to judge whether there are subhealth problems. Therefore, image recognition technology can realize the local analysis of students' physical health and obtain accurate detection data.

## 4. Analysis of Research Results of Students' Physical Health Problems in Class and Exercise Based on Image Recognition Technology

### 4.1. Analysis of Students' Physical Health Changes during Exercise Based on Image Recognition Technology

**Figure 7:**
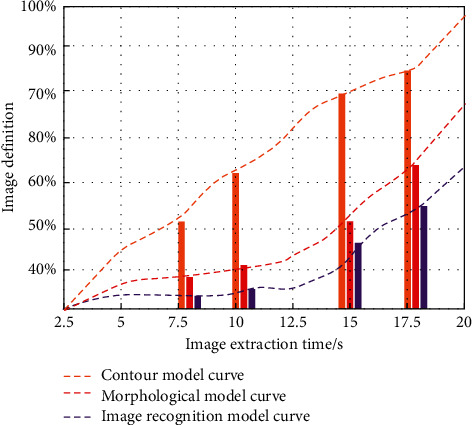
Comparison of image extraction time and definition in three ways.

In the process of tracking and recognizing of students' human action images, based on the students' activity subject and environment model, this paper uses mathematical modeling and classification to construct the structure model of image gray value. Judge the samples of students' moving images in each frame, and finally remove the optimal value according to the peak point to realize the simultaneous recognition, tracking, and classification of multiple targets. Firstly, we use the active contour model, morphological operation model, and image recognition model to compare and analyze. Verify the speed and clarity of the image extraction time of the three methods, and the comparison results are shown in Figure 7.

**Figure 8:**
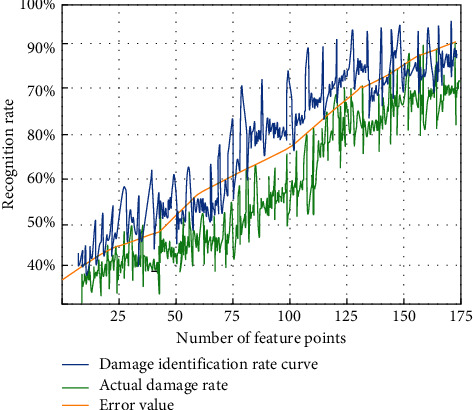
Comparison between damage identification rate and actual damage value.

As can be seen from Figure 7, in the same extraction time change, the image recognition technology has the highest definition for image processing. The average sharpness reaches 98%, while the extraction time of the contour model and morphological model is slow, and the average sharpness is also low. In order to further verify the effect of the recognition method studied in this paper, we compare the student sports injury recognition rate with the actual injury value at the same image feature points, as shown in Figure 8.

It can be seen from Figure 8 that the actual damage value is basically consistent with the identification effect. The image recognition effect can improve the damage range and accuracy to the actual result range and meet the basic requirements of detecting students' physical health.

### 4.2. Analysis of Research Results of Cervical Spine Health Problems in Students' Classroom Activities Based on Image Recognition Technology

**Figure 9:**
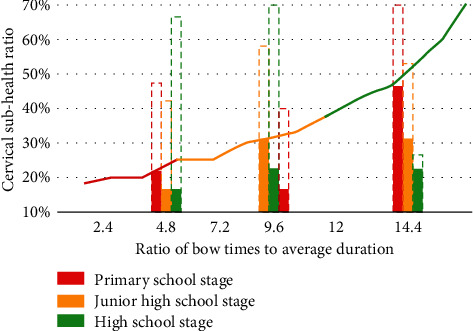
Comparison of the ratio of the number of times of head lowering, the average length of time, and the cervical subhealth ratio of students in the three stages.

In this paper, the same number of students are randomly selected from different schools for experimental research. The ratio of male to female is 1 : 1. The tracking detection is divided into three stages according to age: high school, junior middle school, and primary school. Experiments show that students bow their heads every five minutes in the data feedback obtained by image recognition technology. The average time to look up is 10 to 15 minutes. If calculated at the same time, the number and total intensity of high school students' heads are more than primary and secondary school students. In order to more clearly reflect the changes of the data, we compared the ratio of the number and average duration of head lowering of middle school students in the three stages to the cervical subhealth ratio, as shown in Figure 9.

**Figure 10:**
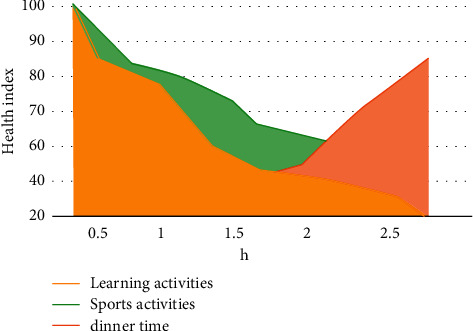
Chart of changes affecting students' physical health.

As can be seen from Figure 9, the ratio of the bow to the duration of students in senior high school changes greatly, and the resulting subhealth problems are more serious. The initial proportion of primary schools is higher, but with the increase of learning time, the learning efficiency of primary school students is significantly reduced. The hidden proportion of high school students is higher, so the subhealth problem is more serious. Finally, according to the image recognition results of students' school activity detection, this paper forms a change curve affecting students' physical health, as shown in Figure 10.

It can be seen from Figure 10 that the horizontal is the time consumed by students in this activity, and the vertical is the change of students' physical health. Most students' physical function and quality are continuously declining in the state of continuous learning. With the increase of physical exercise, their physical health is also improved. The increase of students' meal time can also alleviate the decline of physical function caused by learning. According to the analysis of students' physical health according to their sports and classroom activities, the students' cardiopulmonary function level is general. The failure rate of boys is lower than the overall level, indicating that boys' cardiopulmonary function is better than girls. The overall sensitivity and flexibility of girls are higher than the overall average level. The overall weight change of students is relatively uniform, and a small number of students are underweight and overweight.

## 5. Conclusion

With the continuous emergence of subhealth problems in adolescents, China focuses on the physical health problems of students in the learning stage. Long term study, reading, and writing will lead to an increase in the incidence of the cervical spine and other diseases. Abnormal posture and overtraining in sports will also lead to physical injury. This paper explores and analyzes the changing factors of students' physical health. It mainly uses the image recognition technology to track and recognize the students' action behavior in the process of movement and processes the recognition data in gray scale. Compared with the traditional recognition technology, we use the red mark aggregation region for local capture. This method greatly improves the accuracy and efficiency of image recognition. This paper also pays attention to students' cervical health problems in classroom activities and analyzes the change trend of the cervical spine through students' learning time and low head image capture. Finally, the information data after image recognition is managed and distributed by a graph neural network algorithm. This makes the students' physical health data can be displayed intuitively and can be fed back to the tester more quickly when storing the data. Finally, the experimental results show that most students have physical and subhealth problems. Image recognition technology can quickly extract feature points in the classroom environment and other interference backgrounds, and track and recognize students' behavior targets. Based on the above situation, we need to pay more attention to the healthy development of students. According to the local characteristics of image recognition and analysis, carry out quality education.

## Data Availability

The experimental data used to support the findings of this study are available from the corresponding author upon request.
